# Supplementary material for: Differential stability of task variable representations in retrosplenial cortex
Source: Nat Commun. 2024 Aug 11;15:6872. doi: 10.1038/s41467-024-51227-7 (PMC11316801; doi:10.1038/s41467-024-51227-7)
Supplement: Supplementary file 1 — Supplementary Information [file 41467_2024_51227_MOESM1_ESM.pdf]

SUPPLEMENTAL INFORMATION

Supplementary Fig. 1. Related to Fig. 1.

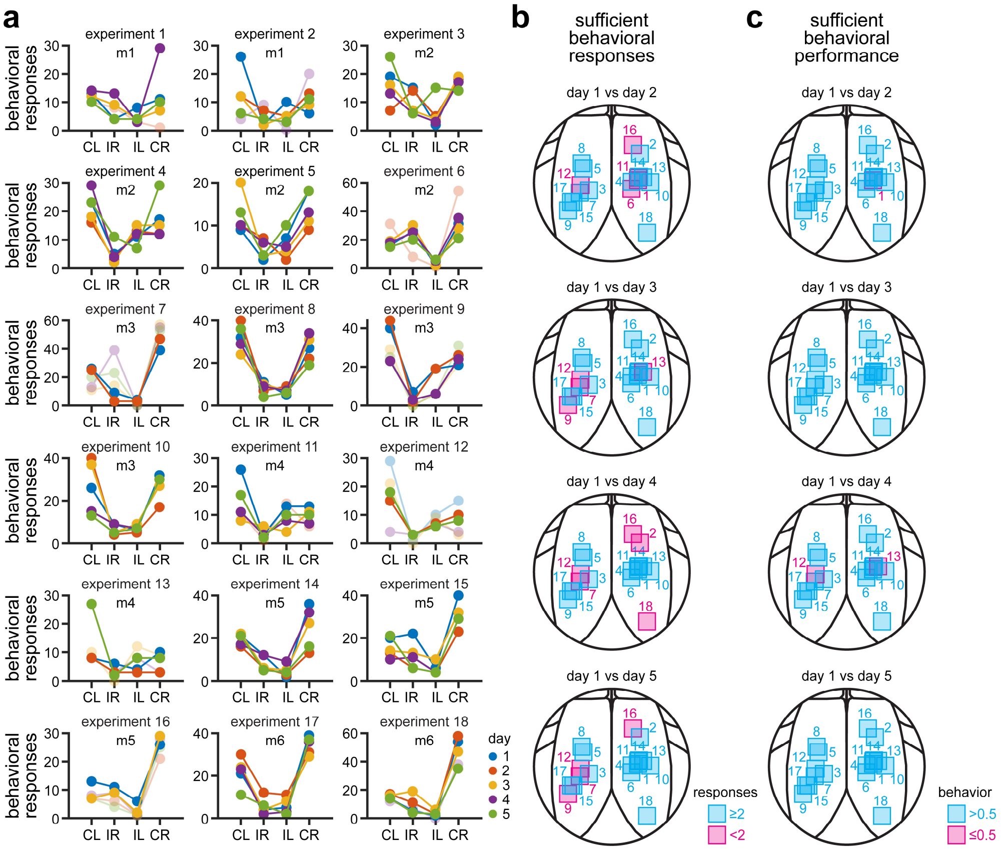

Supplementary Fig. 1. Behavioral responses and location of imaging fields within RSC. Related to Fig. 1.

a. Combinations of correct (C) / incorrect (I) and left (L) / right (R) responses in both contexts across 5 days. Each day is shown in a different color. Blue, day 1; red, day 2; yellow, day 3; purple, day 4; green, day 5. Solid lines show experiments considered for further analyses, whereas faded lines indicate experiments without sufficient behavioral responses or behavioral performance, or both (**Supplementary Fig. 1b**-c).

b-c. Location of imaging fields for all experiments. Fields are color-coded to show experiments included (cyan) and not included (magenta) for analyses according to: (**b**) enough behavioral responses (minimum ≥2 trials of each trial type, see **Supplementary Fig. 1a**), or (**c**) behavioral performance above chance (0.5).

Supplementary Fig. 2. Related to Fig. 1.

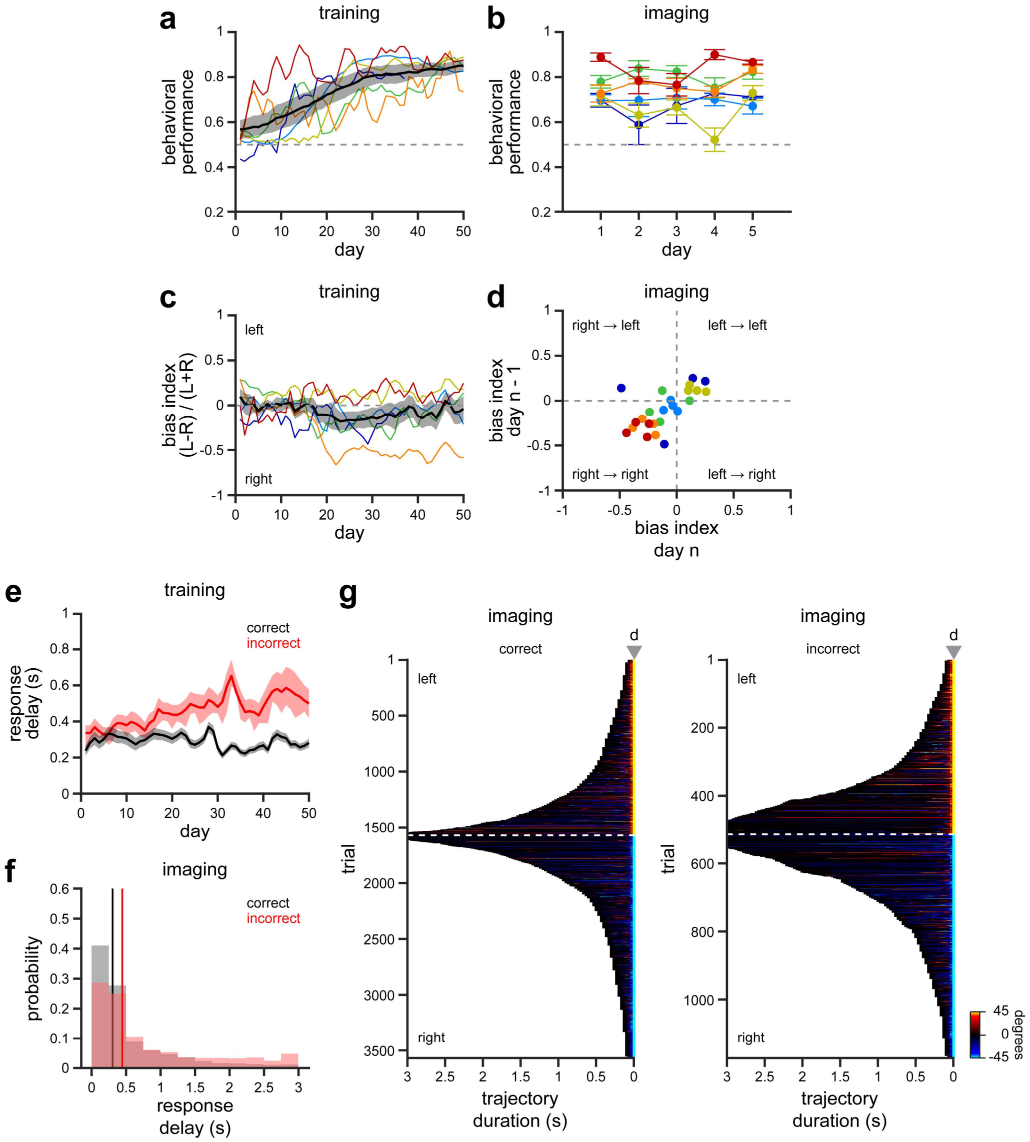

Supplementary Fig. 2. Behavioral parameters during training and imaging sessions. Related to Fig. 1.

a. Behavioral performance during training (n = 6 mice). Performance progressively increased across 50 training sessions until reaching a plateau. Each mouse is shown in a different color. Black solid line, mean across all mice; shaded area, s.e.m.

b. Behavioral performance across 5 consecutive days during imaging sessions. Performance is averaged per mouse (mean ± s.e.m.; n = 18 experiments in 6 mice). Each mouse is shown in a different color.

c. Bias index during training sessions (n = 6 mice). With the exception of one mouse, mice generally perform this task in an unbiased manner, with a similar number of decisions to the left or to the right. Each mouse is shown in a different color. Black solid line, mean across all mice; shaded area, s.e.m.

d. Bias index during imaging sessions. Bias index is averaged per mouse and plotted against its bias index in the previous day (n = 18 experiments in 6 mice). Each mouse is shown in a different color. Response biases during the imaging sessions were small, but consistent across successive days.

e. Decision delay in correct (n = 140-448 trials per day) and incorrect (n = 31-198 trials per day) decisions across all 6 mice during training. Solid line, mean; shaded area, s.e.m. Mice tended to make faster decisions in correct trials by the end of training.

f. Histogram showing the decision delay for correct (n = 1,257) and incorrect (n = 428) trials during imaging sessions across all 6 mice. The median is indicated by a solid line. Mice maintained their faster decisions for correct trials during imaging sessions.

g. Joystick trajectories during decisions in correct (n = 3,735 trials) and incorrect trials (n = 1,225 trials) across all 6 mice. The angle rotation is color-coded as indicated.

Supplementary Fig. 3. Related to Fig. 1.

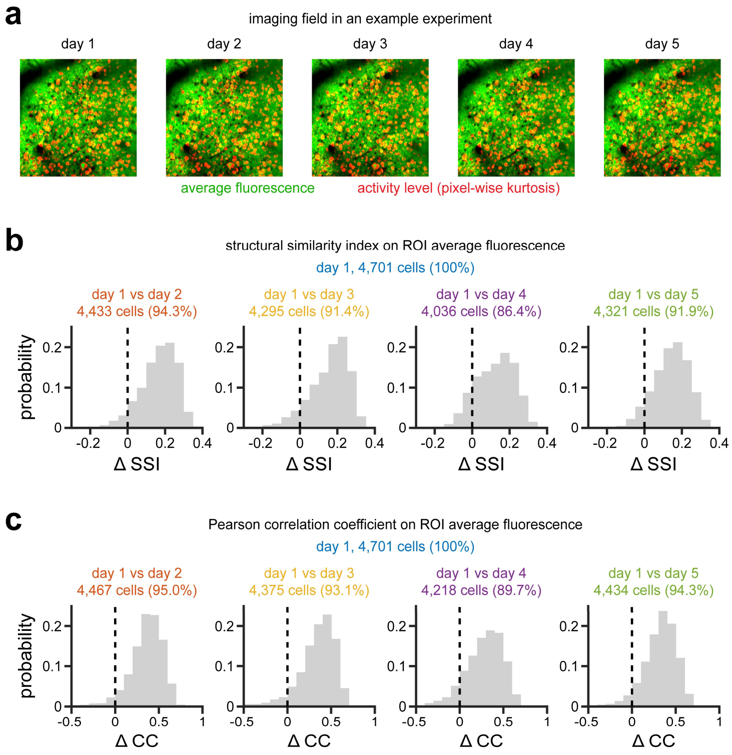

Supplementary Fig. 3. ROI morphological stability across imaging sessions. Related to Fig. 1.

a. Example imaging field (425 × 425 μm) recorded across 5 days. See Figure 1F for examples of individual ROIs.

b. Difference between the structural similarity index (SSI) of each ROI and the 95th CI of their corresponding SSI null distribution (ΔSSI; see **Methods** for details). Only neurons with ΔSSI > 0 were used for subsequent analyses.

c. Similar to **Supplementary Fig. 3b**, only in this case the 2-D correlation coefficient is used to compare the similarity between ROIs. Note that although the 2-D correlation coefficient provides similar results, it is slightly more permissive than the SSI. Thus, we used the SSI as a measure of morphological stability.

Supplementary Fig. 4. Related to Fig. 2.

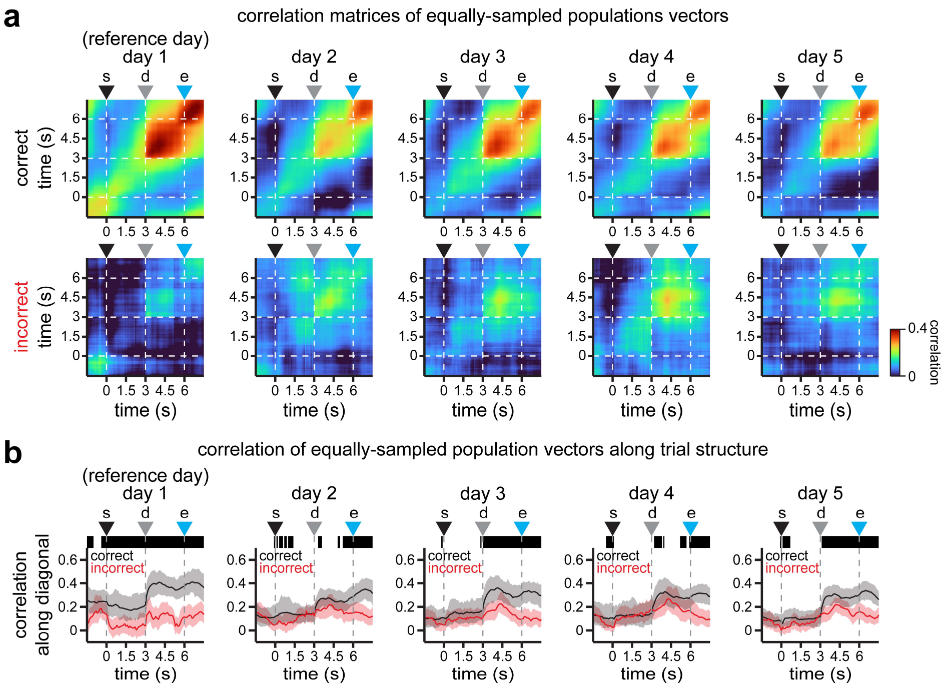

Supplementary Fig. 4. Stability of population activity in equally sampled correct and incorrect trials. Related to Fig. 2.

a. Pearson's correlation coefficient matrices showing the similarity of population activity along trial duration (averaged across 100 iterations). Briefly, population vectors in one half of resampled trials in day 1 are compared to population vectors in the other half of resampled non-overlapping trials in day 1. For other days, all resampled trials in day 1 are compared to all resampled trials in days 2 to 5. In addition, trials are randomly sampled to the minimum number of trials in either correct or incorrect responses for each cell. Moreover, to compare vectors of the same length across days, the population is resampled to 1,609 cells within each iteration (the lowest number of cells that meet criteria in all 5 days). Note the higher similarity of population vectors in correct trials across days, consistent with our findings in the original dataset (**Fig. 2b-c**).

b. Population vector correlation coefficients along the diagonals in **Supplementary Fig. 4a** (n = 100 iterations). Note the higher stability and similarity of population activity in correct trials. Top insets indicate time bins (in black) where the mean correlation in correct trials > 95th CI of the correlation in incorrect trials along trial structure. Solid line, mean; shaded area, 95% CIs.

For all panels: s = trial start; d = decision point; e = trial end.

Supplementary Fig. 5. Related to Fig. 2.

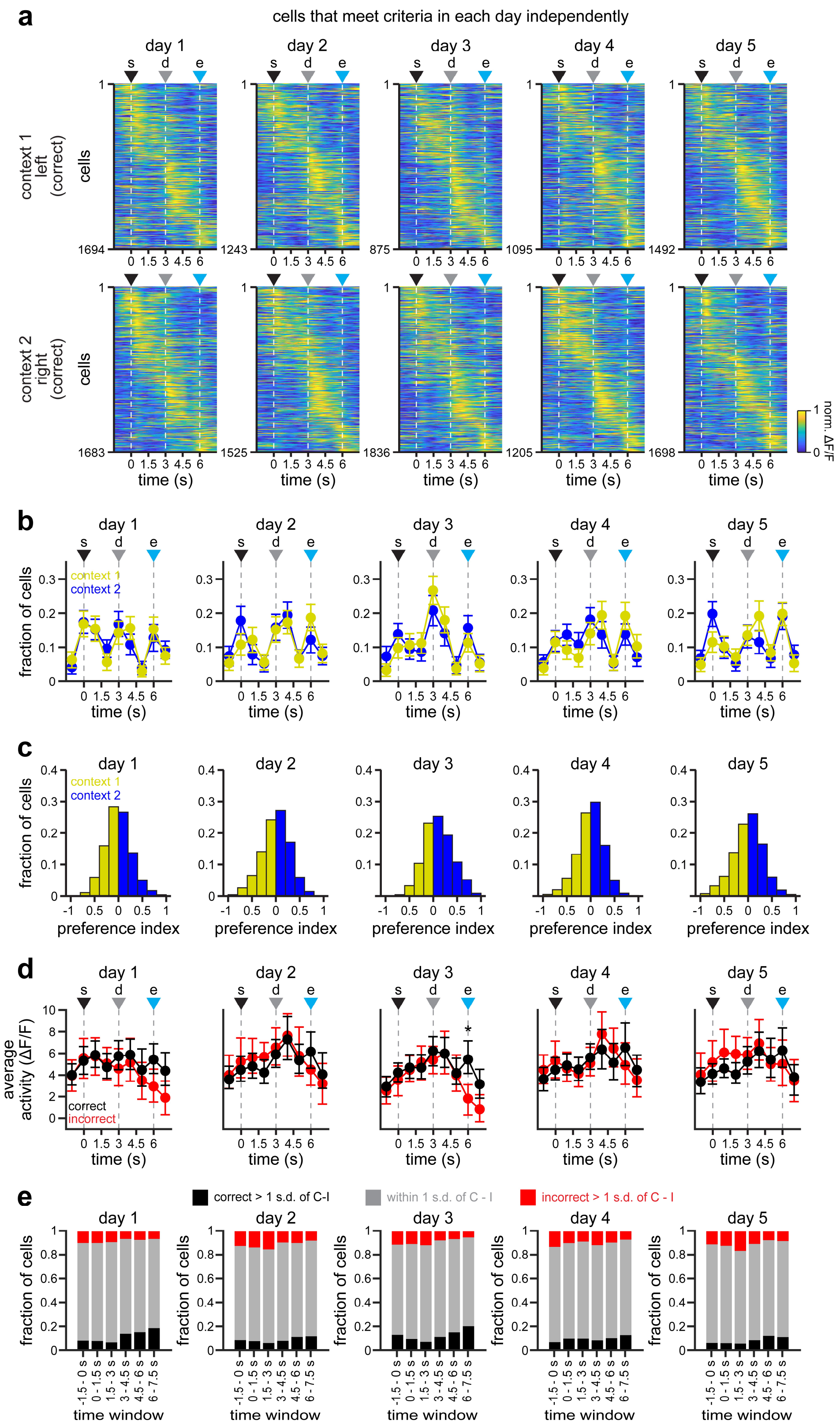

Supplementary Fig. 5. Similar statistics in neural activity on independent days. Related to Fig. 2.

a. Average normalized activity in correct trials for both contexts across the 5 days of experimentation. Responses are cross-validated, sorted in odd trials, and plotted in even trials for each day independently. Note a similar mapping along trial duration by each subpopulation of cells determined in each day independently.

b. Fraction of neurons with peak activity along trial duration for context 1 (yellow) and for context 2 (blue). To estimate population error, the population was randomly sampled (n = 100 neurons per iteration), and the mean ± bootstrapped s.e.m. of the response fraction was plotted.

c. Histograms of the preference index for the cells in **Supplementary Fig. 5a**. Note a similar preference for either context in each population of cells.

d. Average activity in individual neurons for correct and incorrect responses along trial duration. To estimate population error, the population was randomly sampled (n = 100 neurons per iteration), and the mean ± bootstrapped s.e.m. of the average activity was plotted.

e. Fraction of neurons with activity levels within 1 standard deviation of the difference between correct and incorrect (C - I) in gray, and higher that 1 standard deviation in correct (black) and incorrect (red) responses.

For all panels: s = trial start; d = decision point; e = trial end.

Supplementary Fig. 6. Related to Fig. 3.

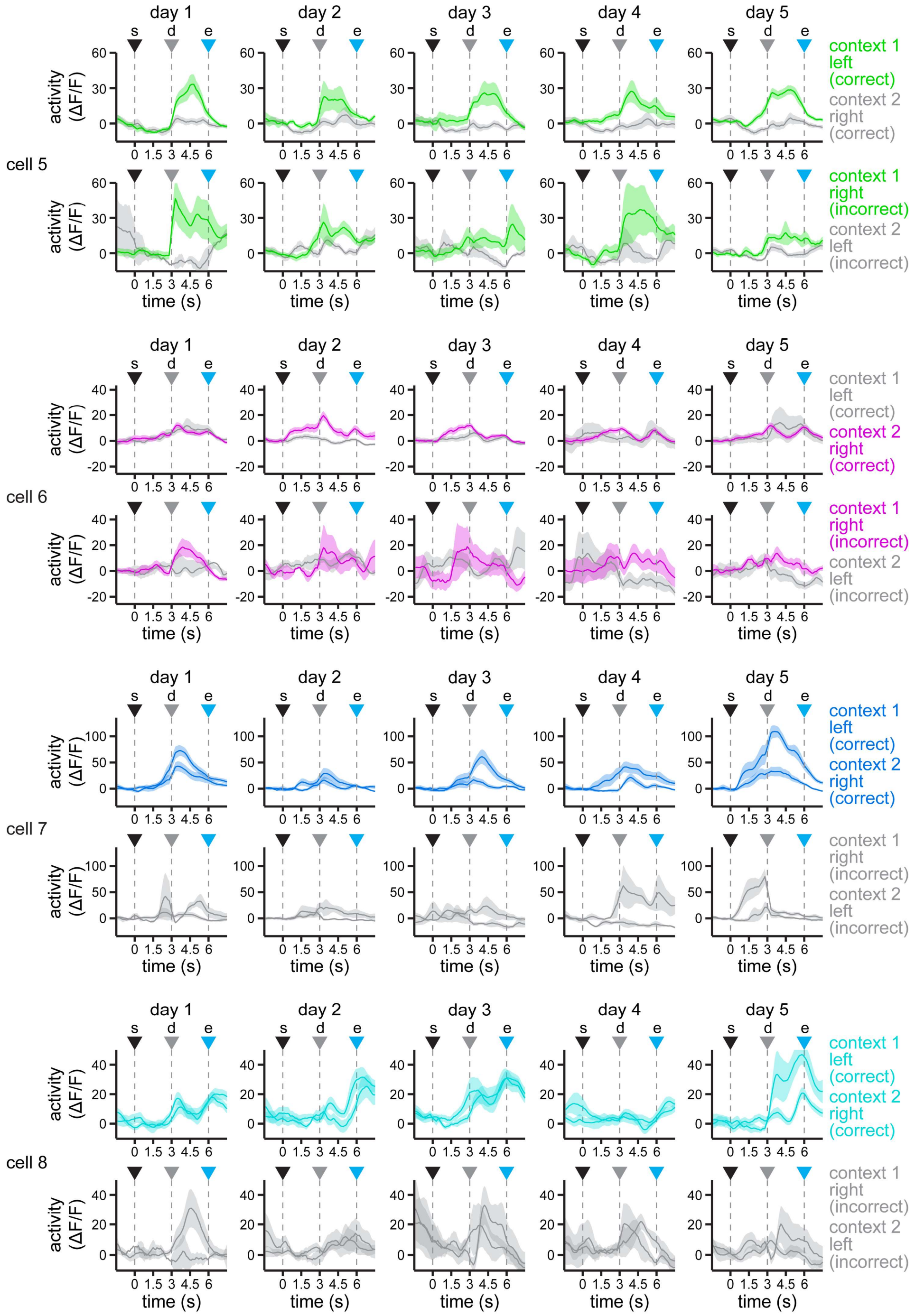

Supplementary Fig. 6. Additional examples of RSC neuron responses during task performance. Related to Fig. 3.

Example neurons exhibiting higher responses for context 1 (cell 5), for right turns (cell 6), and for correct post-decision (cell 7) and post-trial outcomes (cell 8), respectively. Traces are color-coded to highlight the difference between the preferred and non-preferred responses in both correct (top row) and incorrect (bottom row) trials. Solid line, mean; shaded area, s.e.m. s = trial start; d = decision point; e = trial end.

Supplementary Fig. 7. Related to Fig. 4.

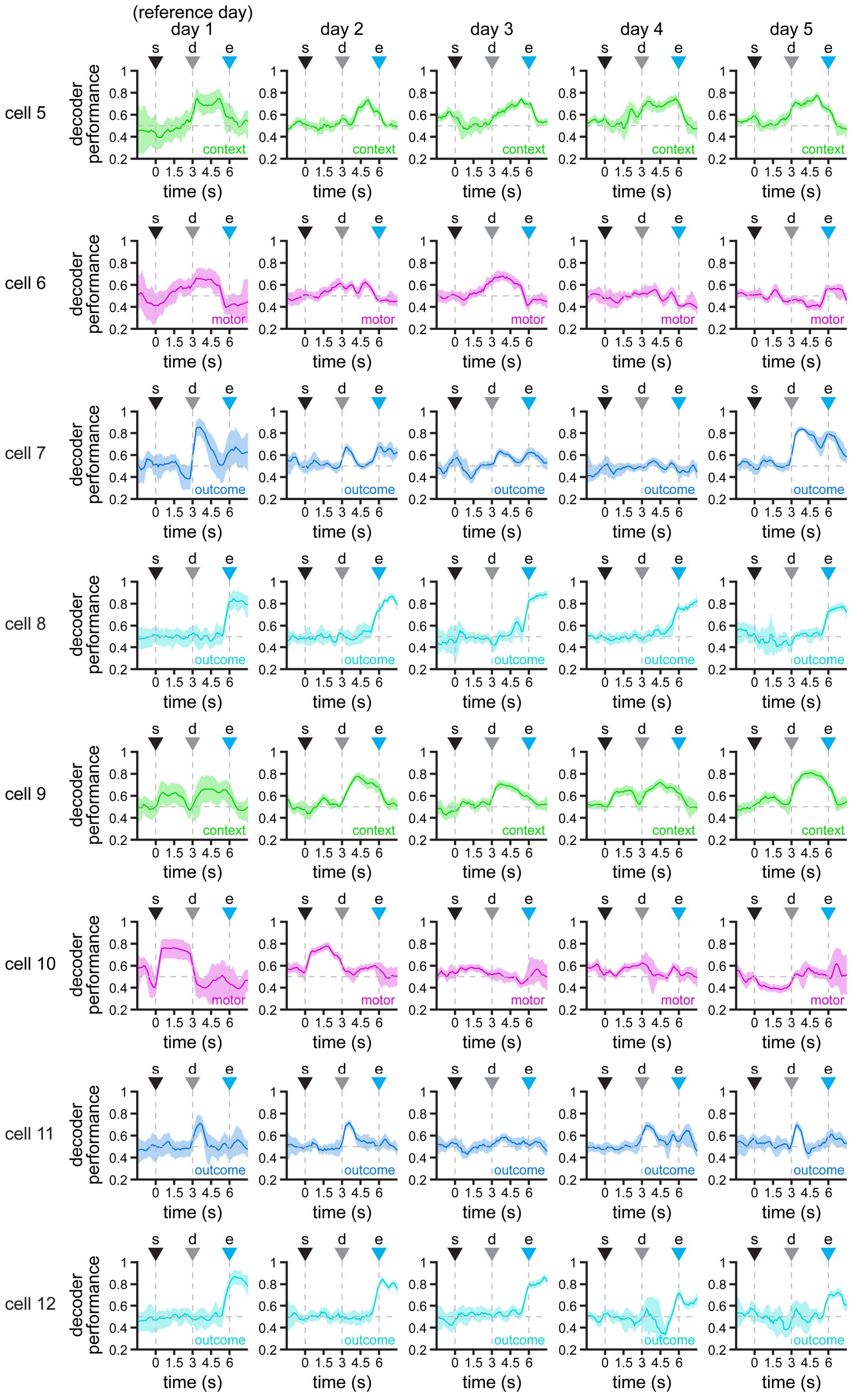

Supplementary Fig. 7. Additional examples of RSC neurons with significant coding of task variables. Related to Fig. 4.

Example cells with significant decoding of context (cells 5 and 9), motor choice (cells 6 and 10), post-decision outcome (cells 7 and 11), and post-trial outcome (cells 8 and 12) in day 1. Solid line, mean; shaded area, 95% CIs. s = trial start; d = decision point; e = trial end.

Supplementary Fig. 8. Related to Fig. 4.

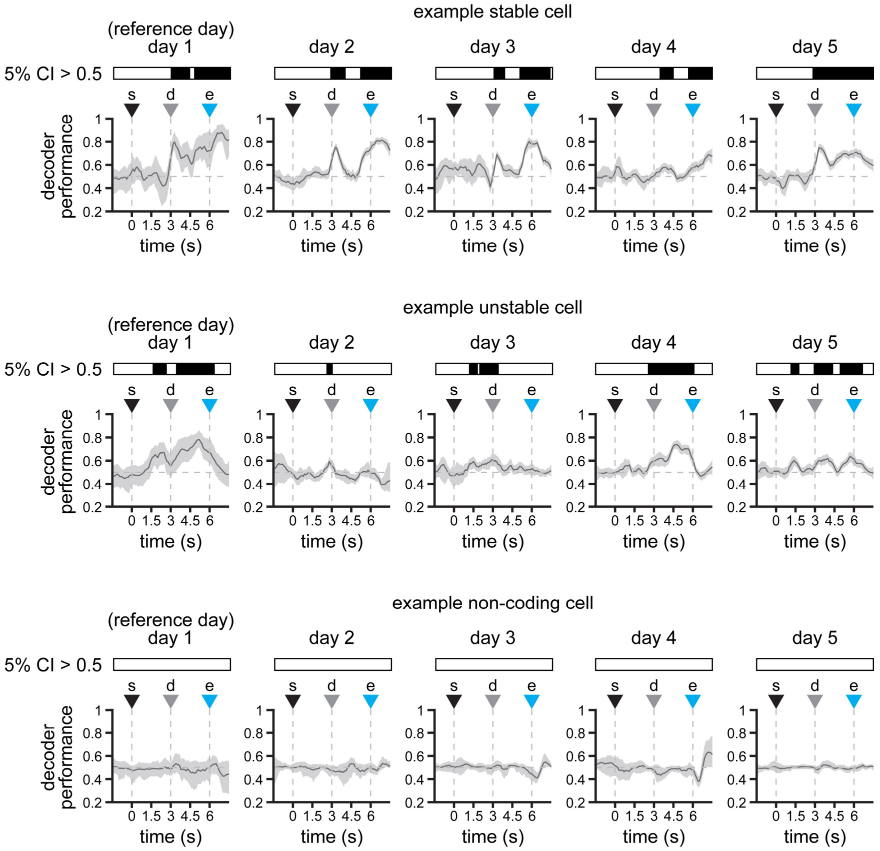

Supplementary Fig. 8. Determination of cells with significant encoding of task variables. Related to Fig. 4. The decoding performance of individual cells is calculated for each time bin by using half of the data for training a support vector machine classifier, and the other half for testing it (see **Methods** for details). We repeat this process multiple times, randomizing the trials used for model training and testing in each iteration. Since we cannot use conventional hypothesis tests with bootstrapped data, the confidence intervals (CI) of decoding performance were used to determine whether a cell exhibited consistent decoding. If the lower 5th CI of decoding performance stayed above chance level (0.5) for 5 consecutive bins, a cell was considered to significantly encode a specific task variable. s = trial start; d = decision point; e = trial end.

Supplementary Fig 9. Related to Fig. 4.

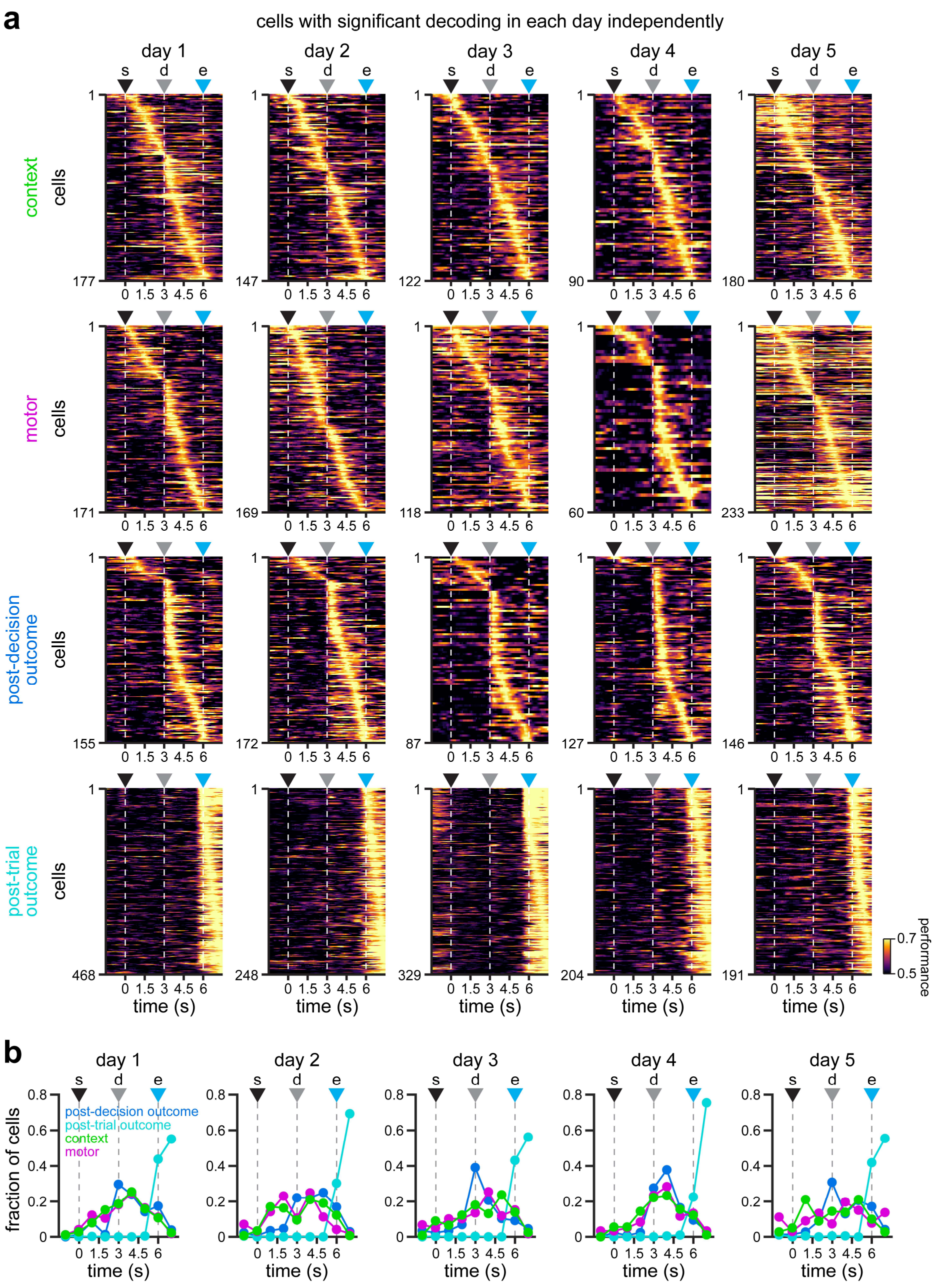

Supplementary Fig. 9. Similar dynamics in the encoding of task variables by individual RSC neurons in each day independently. Related to Fig. 4.

a. Average performance of support vector machine decoders on the activity of cells exhibiting significant decoding of environmental context (top row), motor choice (top-middle row), post-decision outcome (bottom-middle row), and post-trial outcome (bottom row). Models were trained and tested using activity within each day independently. Note similar decoding performance for each task variable in each of the 5 days of experimentation.

b. Fraction of cells exhibiting significant decoding of environmental context, motor choice, post-decision outcome, and post-trial outcome along trial duration. Note similar dynamics for each variable on each independent day.

For all panels: s = trial start; d = decision point; e = trial end.

Supplementary Fig. 10. Related to Fig. 4.

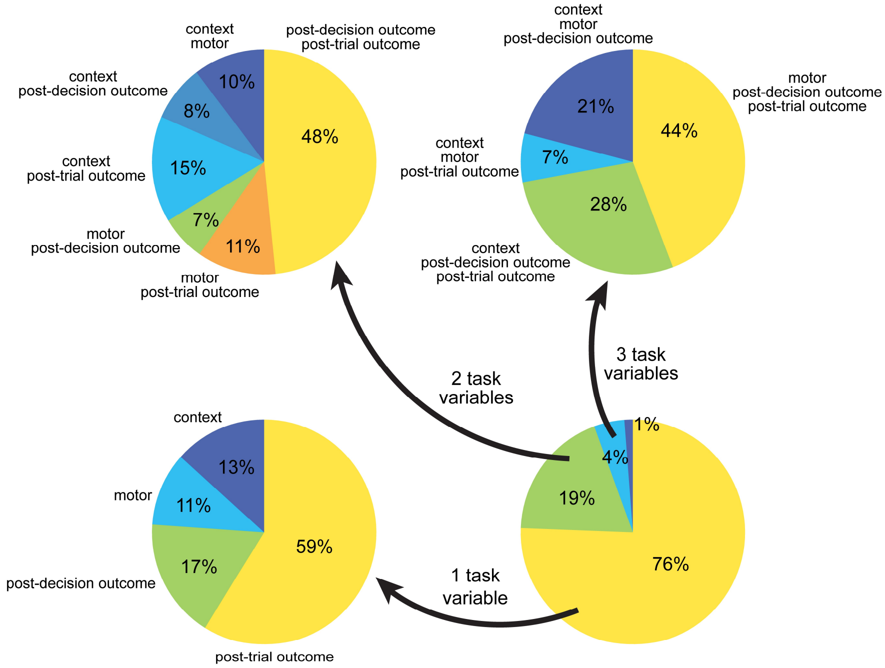

Supplementary Fig. 10. Fraction of RSC neurons with significant decoding of task-variables. Related to Fig. 4.

Fraction of cells with significant encoding of 1, 2, 3, or all 4 task variables (bottom right), broken down by their corresponding fractions when encoding 1 variable (bottom left), 2 variables (top left), and 3 variables (top right).

Supplementary Fig. 11. Related to Fig. 5.

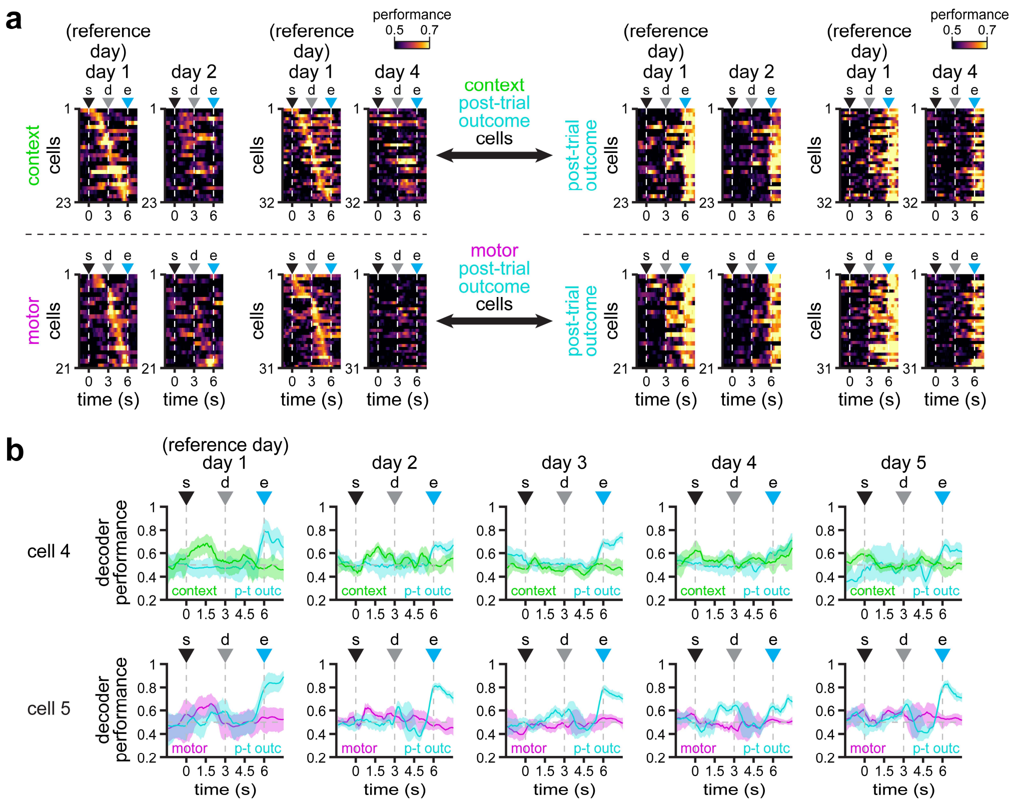

Supplementary Fig. 11. Multiplexing of task information with varying stability within single RSC neurons (additional examples). Related to Fig. 5.

a. Average performance of support vector machine classifiers on the activity of single cells that significantly encode two task variables: context and post-trial outcome (top row), and motor choice and post-trial outcome (bottom row) in day 1. Note the better decoding performance of models trained in day 1 and tested in days 2 and 4 for post-trial outcome.

b. Example cells with significant encoding of context and post-trial outcome (cell 4, top), and motor choice and post-trial outcome (cell 5, bottom) in day 1. Solid line, mean; shaded area, 95% CIs.

For all panels: s = trial start; d = decision point; e = trial end.

Supplementary Fig. 12. Related to Fig. 5.

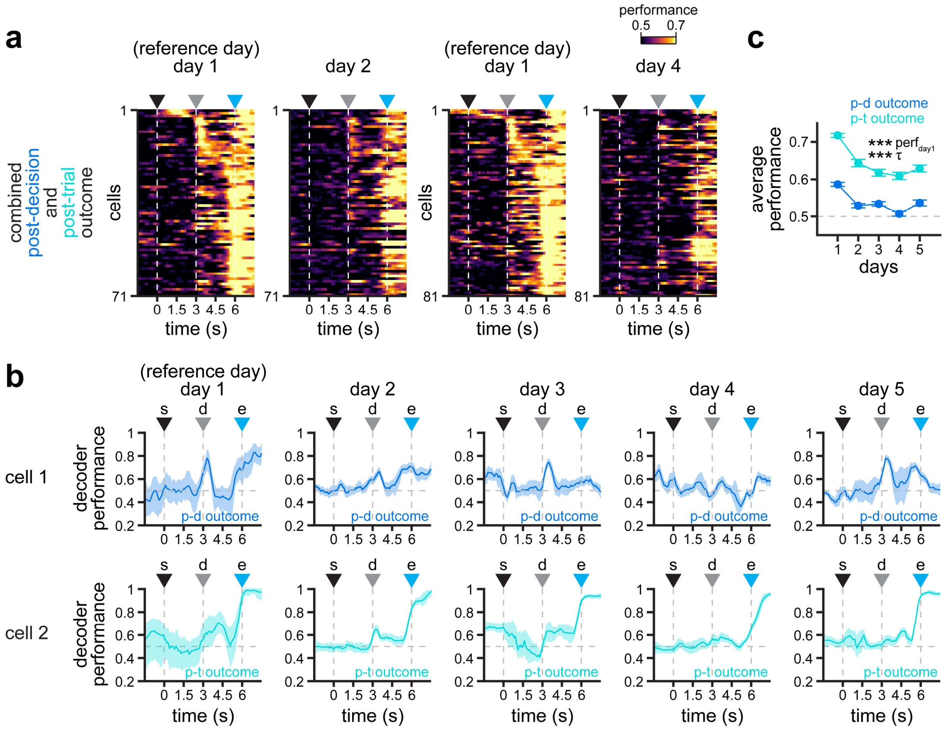

Supplementary Fig. 12. Multiplexing of post-decision and post-trial outcome in individual RSC neurons. Related to Fig. 5.

a. Average performance of support vector machine classifiers on the activity of single cells that significantly encode both post-decision and post-trial outcome in day 1. Note the higher stability of post-trial outcome after trial end.

b. Examples cells with significant encoding of post-decision and post-trial outcome in day 1. Cell 1 (top) exhibits higher stability for post-decision outcome, whereas cell 2 (bottom) exhibits higher stability for post-trial outcome. Solid line, mean; shaded area, 95% CIs.

c. Average decoding performance of post-decision and post-trial outcome within individual cells (mean ± s.e.m.; post-decision outcome -post-trial outcome, days 1-5 = 132, 91, 121, 122, 119 cells). Asterisks denote significantly different decoding performance, as measured by different fit parameters (*p < 0.05, **p < 0.01, ***p < 0.001; LME models; see **Methods** for details). Note the better decoding of the outcome variable after trial end than after the decision point.

For all panels: s = trial start; d = decision point; e = trial end.

Supplementary Fig. 13. Related to Fig. 5.

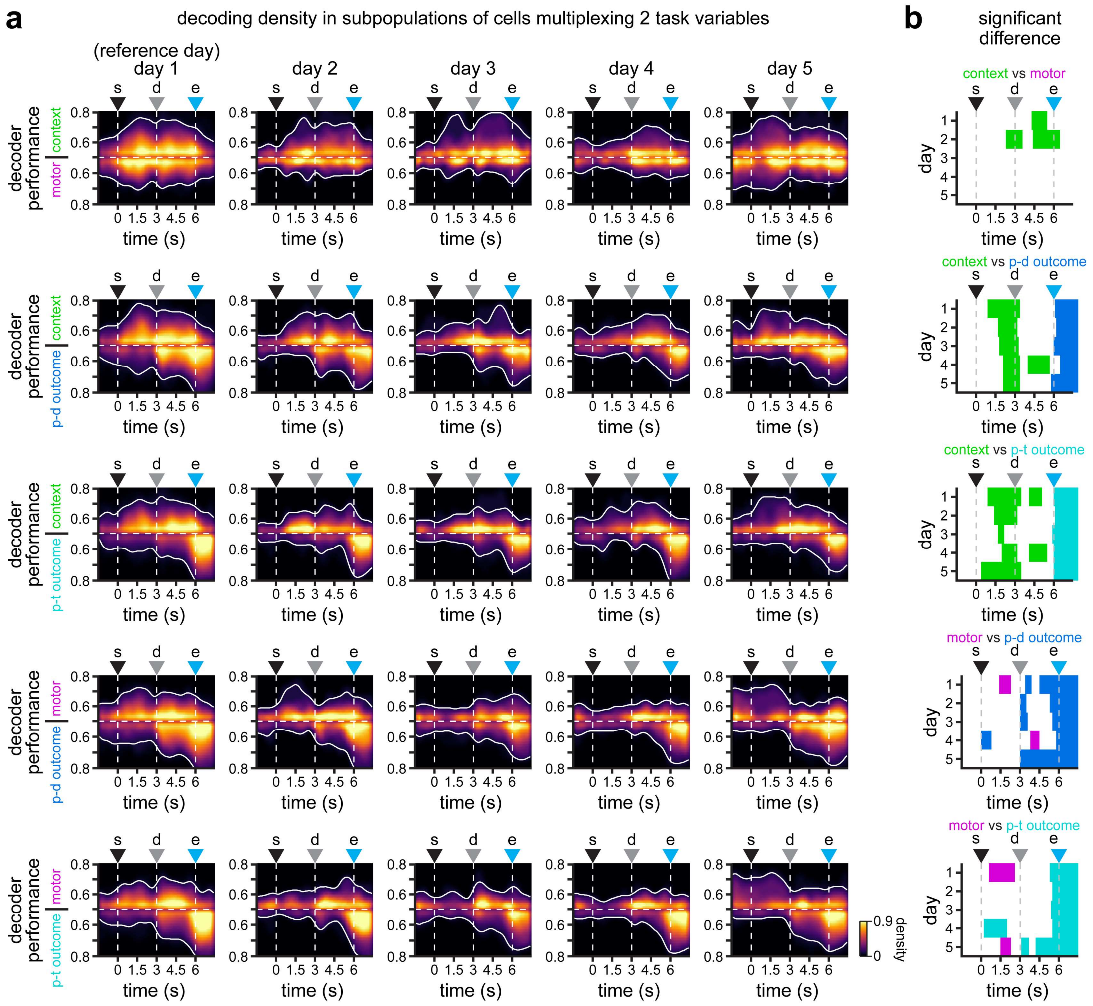

Supplementary Fig. 13. Differential stability in cells with multiplexing encoding of task variables as a function of trial duration. Related to Fig. 5.

a. Heatmaps showing the density of the area under the curve of decoding performance for the subpopulations shown in **Fig. 5a** and **Supplementary Fig. 11**. Task variables are plotted either on top or at the bottom of chance level (0.5), as indicated in the mirrored Y axis. Note the gradient in the stability of single cells multiplexing the indicated task variables, where context and outcome are more stable than motor choice. White lines enclose >10% density.

b. Colormaps showing significant differences between the encoding of task variables indicated in each plot for the subpopulations of cells shown in **Fig. 5a** and **Supplementary Fig. 11** (p < 0.05 for at least 5 consecutive time bins; n = 43 to 132 [day 1], 20 to 71 [day 2], 30 to 88 [day 3], 24 to 81 [day 4], 29 to 93 [day 5] overlapping subpopulation of neurons multiplexing two task variables; LME models). Everything shown in color (not white) is significantly different. Colors indicate the task variable decoded with better performance (green = context; magenta = motor; blue = post-decision outcome, cyan = post-trial outcome).

For all panels: s = trial start; d = decision point; e = trial end.

Supplementary Fig. 14. Related to Fig. 6.

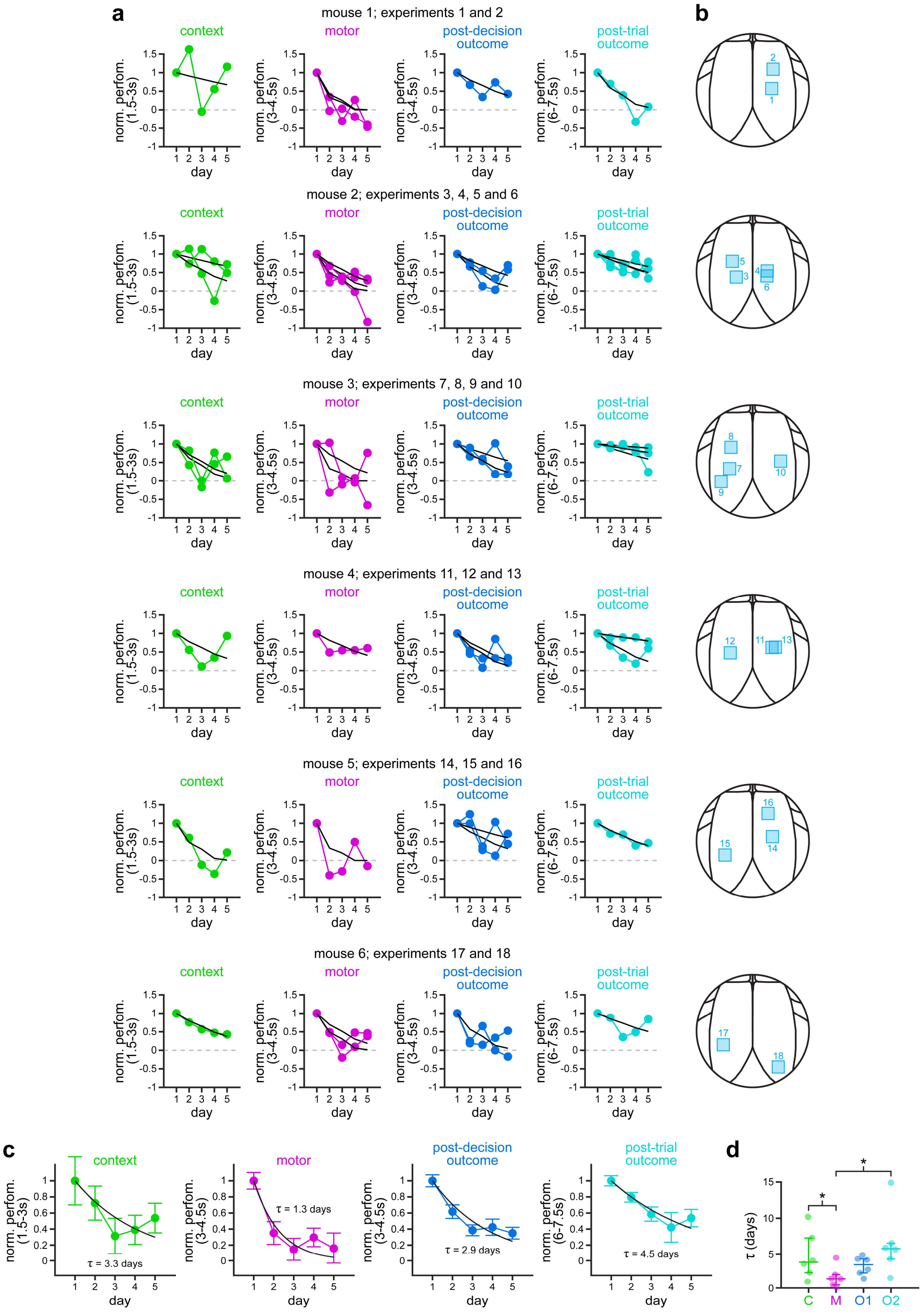

Supplementary Fig. 14. Stability of task variables in individual experiments. Related to Fig. 6.

a. Normalized decoding performance integrated over the indicated 1.5 s windows relevant for each task variable. Exponential decay functions were fit to estimate the decay in encoding stability across days for each individual experiment (black lines). Their corresponding decay constants are plotted in **Fig. 6d**.

b. Maps showing the location of imaging fields within RSC for each mouse.

c. Normalized decoding performance (averaged across mice) integrated over the indicated 1.5 s windows relevant for each task variable (n = 6 mice; mean ± s.e.m.). Exponential decay functions were fit to estimate the decay in encoding stability across days (black lines). Note the faster decay in motor choice and post-decision outcome encoding, and the slower decay in context and post-trial outcome encoding.

d. Time constants (τ) calculated after fitting exponential decay functions to the decoding performance of each task variable in each individual mouse (see **Supplementary Fig. 14a-b**). Dots, individual mice; overlaid lines, median ± 75% CIs (n = 6 mice). Asterisks denote significantly different decay constants (*$p < 0.05$, **$p < 0.01$, ***$p < 0.001$; LME models; see **Methods** for details).

Supplementary Fig. 15. Related to Fig. 6.

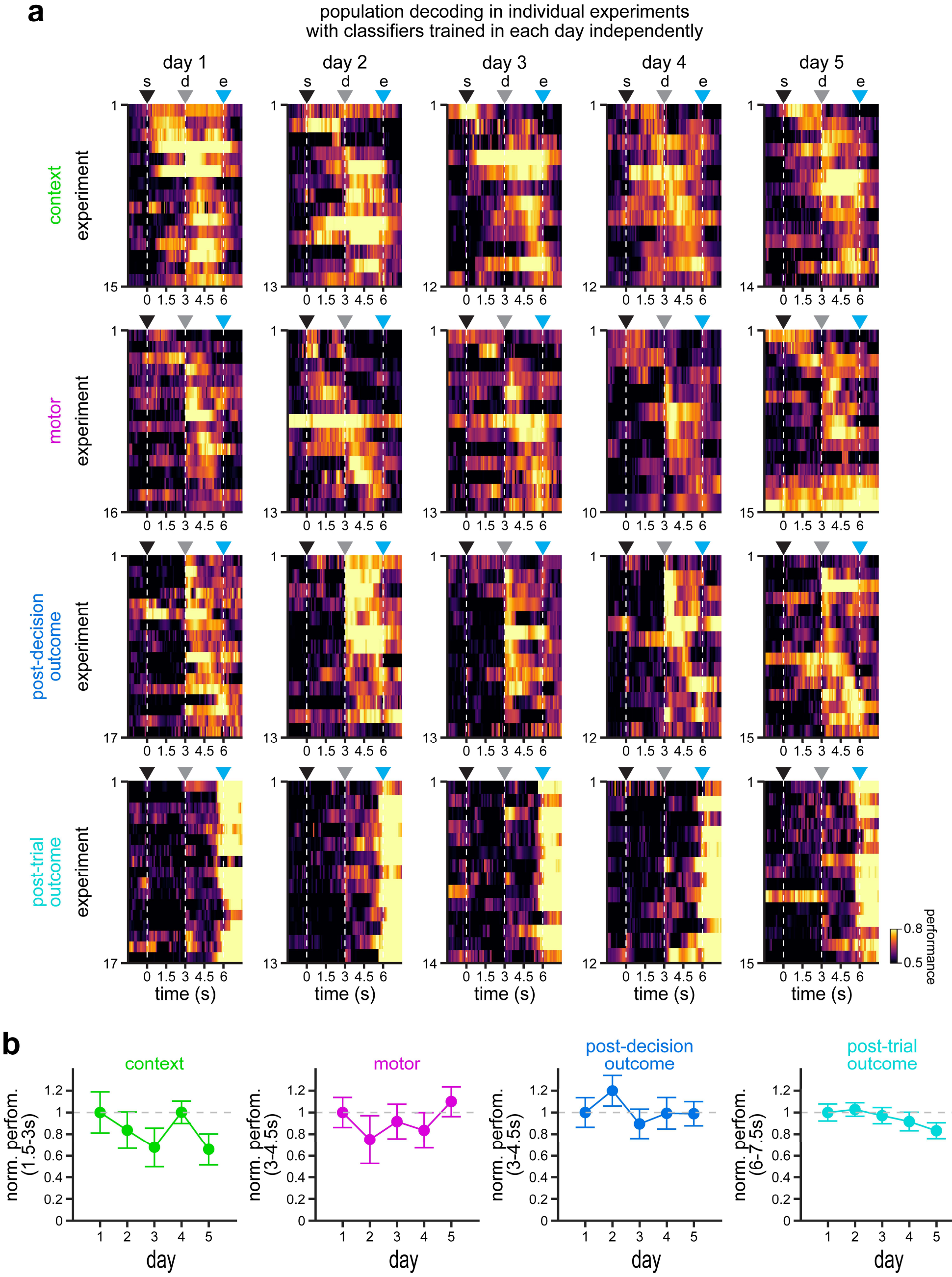

Supplementary Fig. 15. Encoding of task variables in individual experiments. Related to Fig. 6.

a. Average performance of support vector machine classifiers on the activity of different subsets of cells separated by experiment: context (top row), motor choice (top-middle row), post-decision outcome (bottom-middle row), and post-trial outcome (bottom row). Models were trained and tested using activity within each day independently. Note similar decoding performance for each task variable in each of the 5 days of experimentation.

b. Normalized decoding performance integrated over the indicated 1.5 s windows relevant for each task variable (mean ± bootstrapped s.e.m. across experiments; n = 100 iterations). Note how decoding performance remains largely unchanged across all days.

For all panels: s = trial start; d = decision point; e = trial end.

Supplementary Fig. 16. Related to Fig. 7.

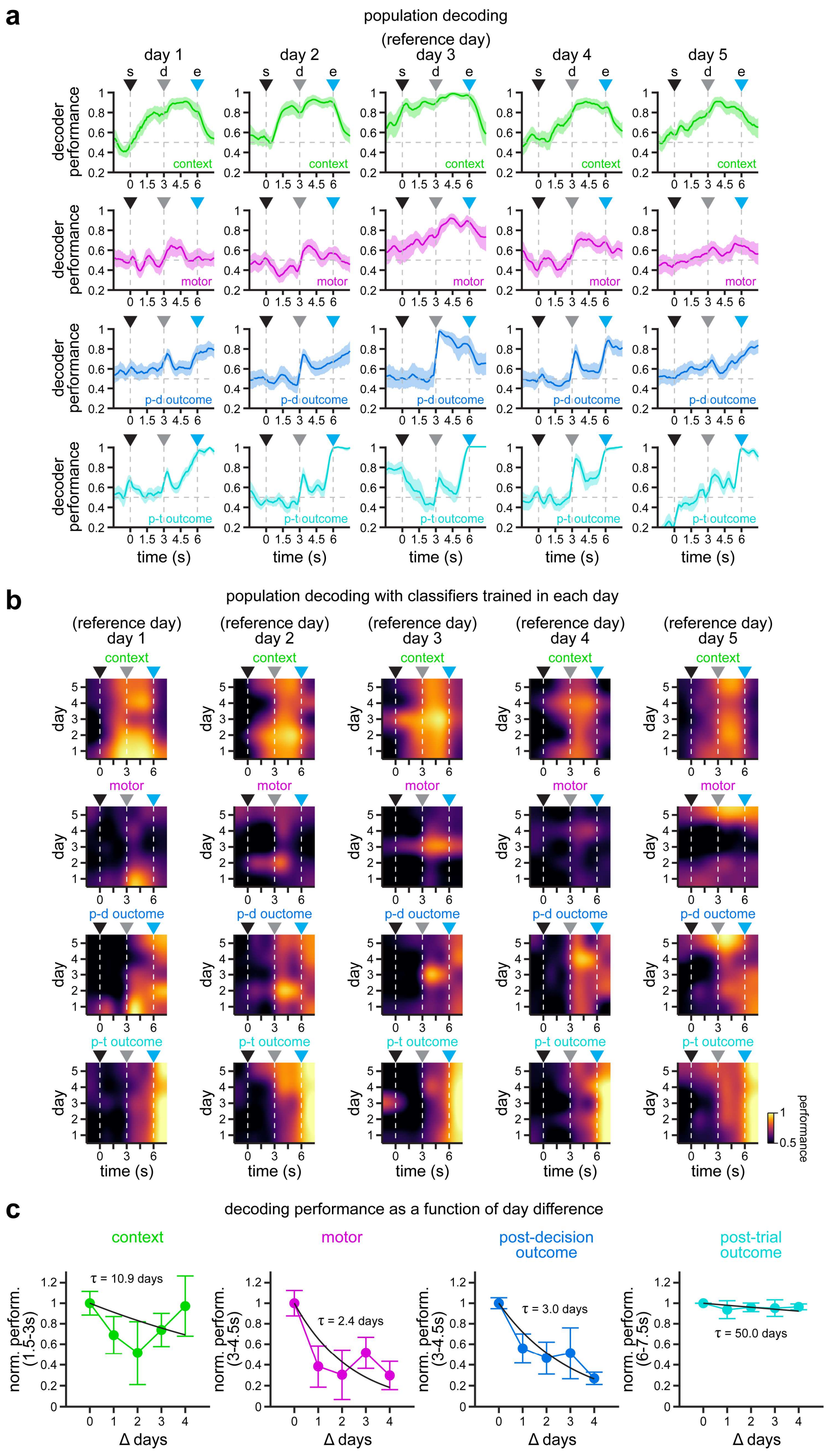

Supplementary Fig. 16. Differential stability in population encoding of task variables in the RSC (classifiers trained in each day independently). Related to Fig. 7.

a. Encoding of context (top row), motor choice (top-middle row), post-decision outcome (bottom-middle row) and post-trial outcome (bottom row) by all recorded RSC neurons across all mice using day 3 as reference. Solid line, mean; shaded area, 95% CIs. Note the gradient in population encoding, where post-trial outcome and context are more stable than post-decision outcome and motor choice.

b. Heat maps of context (top row), motor choice (top-middle row), post-decision outcome (bottom-middle row), and post-trial outcome (bottom row) coding performance along trial duration and across all 5 days of experimentation when different days are used as reference. Note that the results are consistent regardless of the reference day used for training our support vector machine classifiers. Namely, higher stability of context and outcome variables, and lower stability of motor choice.

c. Normalized decoding performance integrated over the indicated 1.5 s windows relevant for each task variable (mean ± bootstrapped s.e.m.; n = 100 iterations) as a function of day difference between the training of the classifier and testing on past and future days. Exponential decay functions were fit to estimate the decay in encoding stability across days (black lines denote fits to the average normalized performance). Note the faster decay in motor choice and post-decision outcome encoding, and the slower decay in context and post-trial outcome encoding. To obtain CIs for the decay constants, we also fit decay functions to each iteration of the decoder performance (95% CIs, context = 4.6 – 50.0 days, motor = 0.7 – 3.9 days, post-decision outcome = 1.7 – 4.3 days, post-trial outcome = 26.7 – 50.0 days).

For all panels: s = trial start; d = decision point; e = trial end.

Supplementary Fig. 17. Related to Fig. 7.

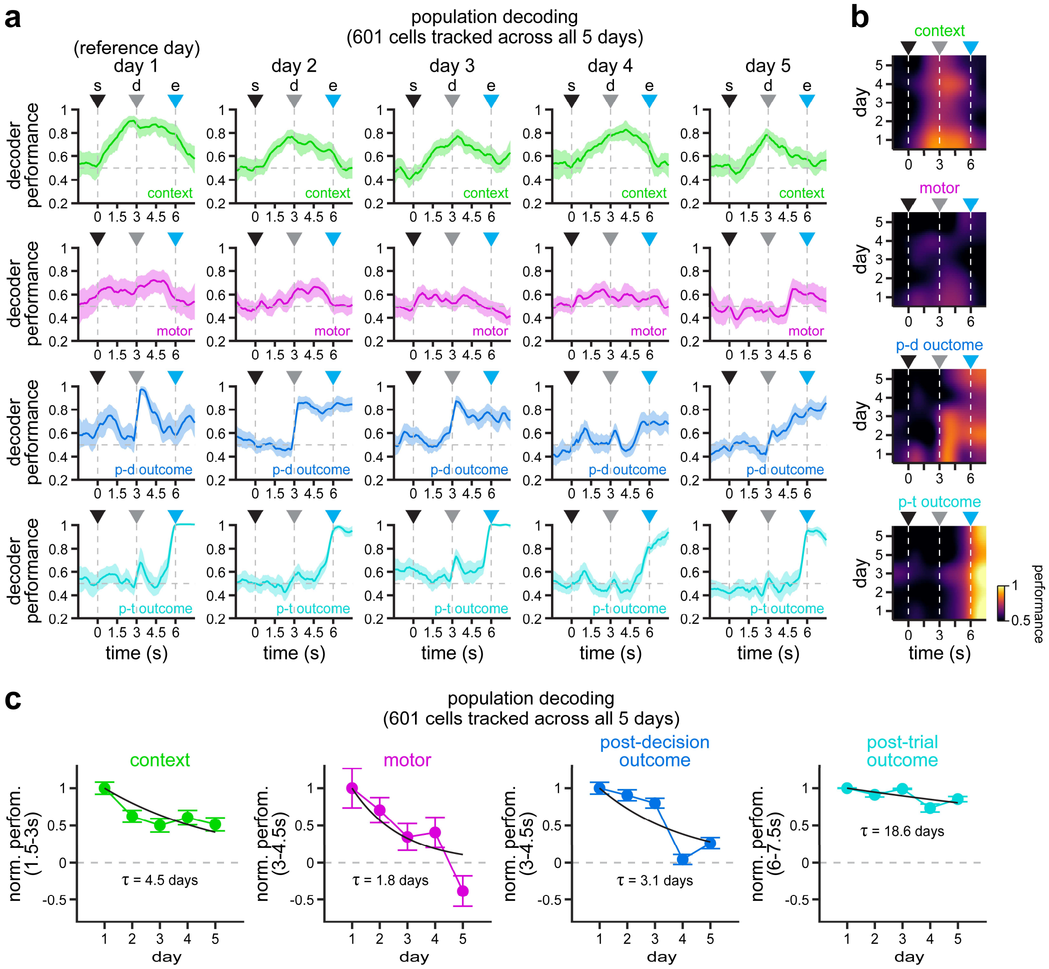

Supplementary Fig. 17. Population encoding of task variables by a subpopulation of RSC neurons that can be tracked consecutively across 5 days. Related to Fig. 7.

a. Encoding of context (top row), motor choice (top-middle row), post-decision outcome (bottom-middle row) and post-trial outcome (bottom row) by the 601 cells that pass all tests in all days for inclusion in our analyses (ROI morphological stability, behavior criteria, and activity reliability). Solid line, mean; shaded area, 95% CIs. Note a similar gradient in population encoding as the one observed with the larger population of cells (**Fig. 7a**).

b. Heat maps of context (top), motor choice (top-middle), post-decision outcome (bottom-middle), and post-trial outcome (bottom) coding performance along trial duration and across all 5 days of experimentation. Note the similar dynamics in the encoding and stability of the different task variables (**Fig. 7b**).

c. Normalized decoding performance integrated over the indicated 1.5 s windows relevant for each task variable (mean ± bootstrapped s.e.m.; n = 100 iterations). Exponential decay functions were fit to estimate the decay in encoding stability across days (black lines denote fits to the average normalized performance). Again, note similar dynamics (**Fig. 7c**), with a faster decay in motor choice and post-decision outcome encoding, and a slower decay in context and post-trial outcome encoding. To obtain CIs for the decay constants, we also fit decay functions to each iteration of the decoder performance (95% CIs, context = 3.4 – 5.9 days, motor = 1.2 – 2.5 days, post-decision outcome = 2.7 – 3.7 days, post-trial outcome = 15.0 – 23.9 days).

For all panels: s = trial start; d = decision point; e = trial end.

Supplementary Fig. 18. Related to Fig. 7.

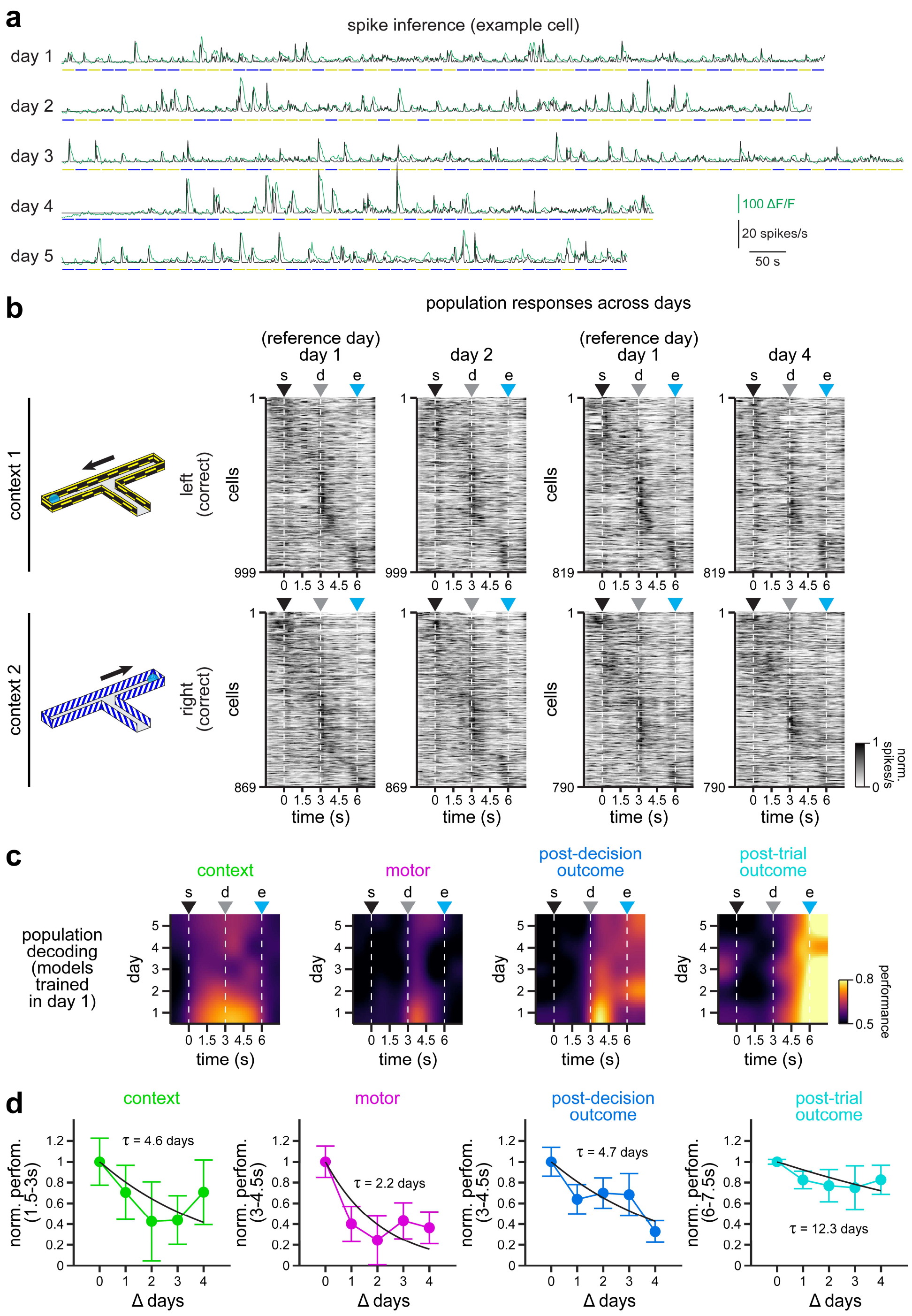

Supplementary Fig. 18. Classifiers trained on inferred spikes reveal similar dynamics in the stability of task-related representations in the RSC. Related to Fig. 7.

a. Overlay of calcium signals (green) and inferred spike traces (black) for an example cell. Spikes were estimated by a sparse, nonnegative convolution algorithm (OASIS) with an autoregressive model of order 1 for the convolution kernel[60].

b. Averaged normalized spikes in the population of neurons separated by their preferred context. Only comparisons between days 1 and 2, and days 1 and 4 are shown for clarity. Spikes are cross-validated, sorted by latency using odd trials in day 1, and plotted using even trials for days 2 or 4.
c. Heat maps of context (left), motor choice (middle-left), post-decision outcome (middle-right), and post-trial outcome (right) coding performance along trial duration and across all 5 days of experimentation. Note the similar dynamics in the encoding and stability of task variables across days (**Fig. 7b**), even when estimated from inferred spikes.
d. Normalized decoding performance integrated over the indicated 1.5 s windows relevant for each task variable (mean ± bootstrapped s.e.m.; n = 100 iterations) as a function of day difference between the training of the classifier and testing in past and future days. Exponential decay functions were fit to estimate the decay in encoding stability across days (black lines denote fits to the average normalized performance). Note similar dynamics, with a faster decay in motor choice and post-decision outcome encoding, and a slower decay in context and post-trial outcome encoding. To obtain CIs for the decay constants, we also fit decay functions to each iteration of the decoder performance (95% CIs, context = 1.3 – 13.1 days, motor = 0.6 – 3.8 days, post-decision outcome = 2.8 – 7.0 days, post-trial outcome = 6.4 – 40.5 days).
For all panels: s = trial start; d = decision point; e = trial end.

Supplementary Fig. 19. Related to Fig. 7.

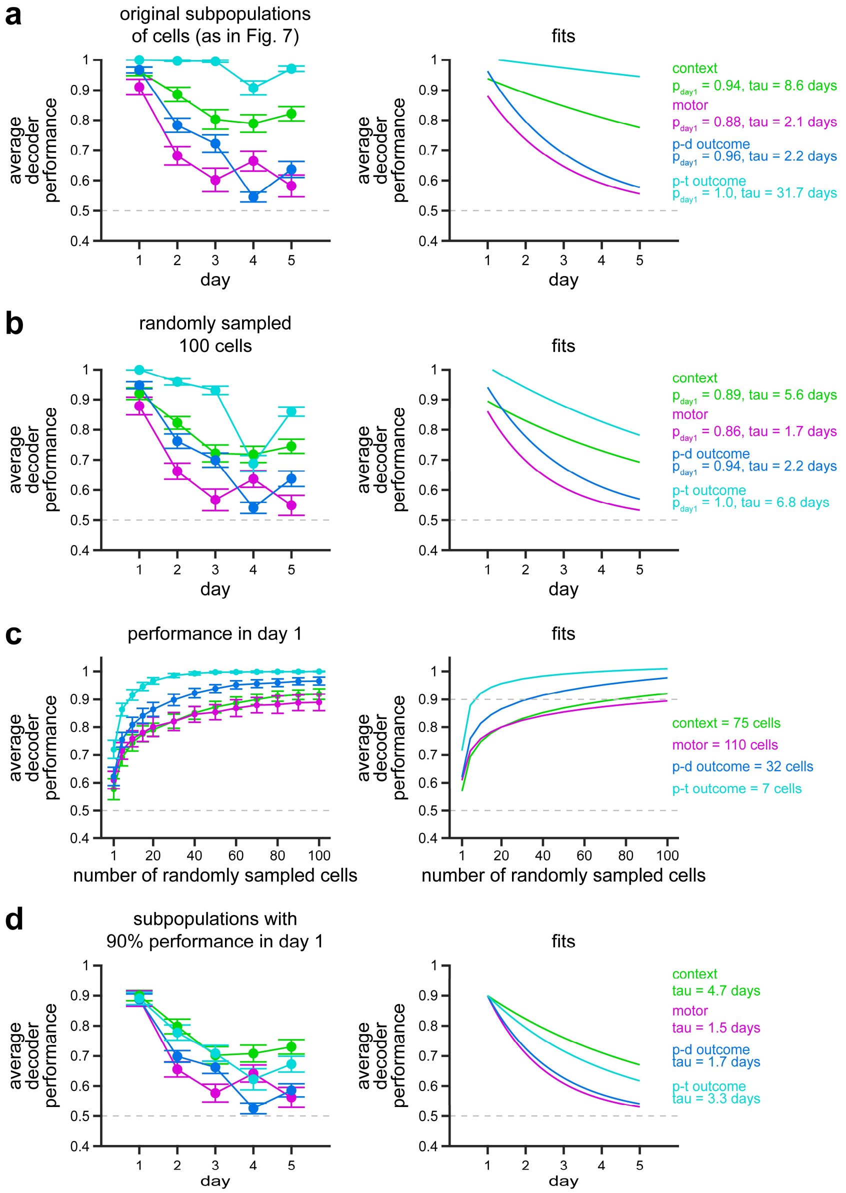

Supplementary Fig. 19. Average decoding performance of classifiers trained on each day independently. Related to Fig. 7.

a. Average decoding performance over the indicated 1.5 s windows relevant for each task variable across days (mean ± bootstrapped s.e.m.; n = 100 iterations). Note the faster decay in decoder performance for motor and post-decision outcome. By contrast, context and post-trial outcome show higher stability. Fits are shown on the right, with their corresponding values for performance in day 1 as well as their decay constants.

b. Similar to A, only in this case, subpopulations were resampled to 100 neurons in each iteration during training and testing of classifiers (mean ± bootstrapped s.e.m.; n = 100 iterations). This ensures all task variables are evaluated with an identical population size. Note a similar trend, where context and post-trial outcome exhibit higher stability. Fits are shown on the right, with their corresponding values for performance in day 1 as well as their decay constants.

c. Average decoding performance for randomly sampled neurons over 100 iterations in day 1 (mean ± bootstrapped s.e.m.; n = 100 iterations). The decoder performance (P) as a function of the number of neurons (n) was fit to a power function ($\mathrm{P} = a * n^b + c$), and the number of neurons corresponding to 90% decoding performance in day 1 was estimated, as indicated.

d. Similar to A, but with different subpopulations of sampled neurons corresponding to 90% decoding performance in day 1 (as in **C;** mean ± bootstrapped s.e.m.; n = 100 iterations). Note the higher stability of context and post-trial outcome, and the lower stability of motor and post-decision outcome.
