# Supplementary material for: Differential stability of task variable representations in retrosplenial cortex
Source: Nat Commun. 2024 Aug 11;15:6872. doi: 10.1038/s41467-024-51227-7 (PMC11316801; doi:10.1038/s41467-024-51227-7)
Supplement: Supplementary file 3 — Description of additional supplementary files [file 41467_2024_51227_MOESM3_ESM.pdf]

<u>Description of Additional Supplementary files</u>

Supplementary Movie 1. - Calcium imaging and behavior videos from the same mouse over several sessions (Day 1: upper left; Day 3: upper right; Day 4: lower left; Day 5: lower right). Each trial type is annotated.
