# Supplementary material for: Differential stability of task variable representations in retrosplenial cortex
Source: Nat Commun. 2024 Aug 11;15:6872. doi: 10.1038/s41467-024-51227-7 (PMC11316801; doi:10.1038/s41467-024-51227-7)
Supplement: Supplementary file 5 — Reporting Summary [file 41467_2024_51227_MOESM5_ESM.pdf]

nature portfolio

Corresponding author(s): Luis Franco & Michael Goard

Last updated by author(s): Jul 20, 2024

Reporting Summary

Nature Portfolio wishes to improve the reproducibility of the work that we publish. This form provides structure for consistency and transparency in reporting. For further information on Nature Portfolio policies, see our Editorial Policies and the Editorial Policy Checklist.

Statistics

For all statistical analyses, confirm that the following items are present in the figure legend, table legend, main text, or Methods section.

n/a	Confirmed	
☐	☒	The exact sample size (*n*) for each experimental group/condition, given as a discrete number and unit of measurement
☐	☒	A statement on whether measurements were taken from distinct samples or whether the same sample was measured repeatedly
☐	☒	The statistical test(s) used AND whether they are one- or two-sided<br>*Only common tests should be described solely by name; describe more complex techniques in the Methods section.*
☒	☐	A description of all covariates tested
☐	☒	A description of any assumptions or corrections, such as tests of normality and adjustment for multiple comparisons
☐	☒	A full description of the statistical parameters including central tendency (e.g. means) or other basic estimates (e.g. regression coefficient) AND variation (e.g. standard deviation) or associated estimates of uncertainty (e.g. confidence intervals)
☐	☒	For null hypothesis testing, the test statistic (e.g. F, t, r) with confidence intervals, effect sizes, degrees of freedom and P value noted<br>*Give P values as exact values whenever suitable.*
☒	☐	For Bayesian analysis, information on the choice of priors and Markov chain Monte Carlo settings
☒	☐	For hierarchical and complex designs, identification of the appropriate level for tests and full reporting of outcomes
☒	☐	Estimates of effect sizes (e.g. Cohen's *d*, Pearson's *r*), indicating how they were calculated

Our web collection on statistics for biologists contains articles on many of the points above.

Software and code

Policy information about availability of computer code

Data collection	Matlab 2017a (Mathworks) and above versions were used for acquisition of behavioral data. Prairie View V5.4 (Bruker) was used for acquisition of two-photon imaging data.
Data analysis	All analyses were carried out in Matlab 2017a (Mathworks) and above versions. Finalized codes will be deposited in Github upon acceptance of the manuscript.

For manuscripts utilizing custom algorithms or software that are central to the research but not yet described in published literature, software must be made available to editors and reviewers. We strongly encourage code deposition in a community repository (e.g. GitHub). See the Nature Portfolio guidelines for submitting code & software for further information.

Data

Policy information about availability of data

All manuscripts must include a data availability statement. This statement should provide the following information, where applicable:

- Accession codes, unique identifiers, or web links for publicly available datasets
- A description of any restrictions on data availability
- For clinical datasets or third party data, please ensure that the statement adheres to our policy

All processed calcium imaging data used in this study will be deposited in Dryad upon acceptance of the manuscript.

Human research participants

Policy information about studies involving human research participants and Sex and Gender in Research.

Reporting on sex and gender	n/a
Population characteristics	n/a
Recruitment	n/a
Ethics oversight	n/a

Note that full information on the approval of the study protocol must also be provided in the manuscript.

Field-specific reporting

Please select the one below that is the best fit for your research. If you are not sure, read the appropriate sections before making your selection.

☒ Life sciences ☐ Behavioural & social sciences ☐ Ecological, evolutionary & environmental sciences

For a reference copy of the document with all sections, see nature.com/documents/nr-reporting-summary-flat.pdf

Life sciences study design

All studies must disclose on these points even when the disclosure is negative.

Sample size	No a priori sample size calculation was performed. Sample sizes were chosen based on previous similar studies (n = 5-day 18 experiments in 6 mice).
Data exclusions	Some data excluded for analyses are detailed in Fig. S1. The rationale for this is the lack of incorrect behavioral responses on specific days.
Replication	Experiments were repeated in multiple mice. Findings are not exclusive to any specific mouse.
Randomization	All mice were tested in identical conditions. No randomization of mice was necessary for this study. For some analyses, neuronal responses were grouped according to their preference to a specific task variable.
Blinding	For analyses of neuronal responses, investigators were not blinded to group allocation. Group allocation was determined after calculating response preference.

Reporting for specific materials, systems and methods

We require information from authors about some types of materials, experimental systems and methods used in many studies. Here, indicate whether each material, system or method listed is relevant to your study. If you are not sure if a list item applies to your research, read the appropriate section before selecting a response.

Materials & experimental systems

n/a	Involved in the study	
☒	☐	Antibodies
☒	☐	Eukaryotic cell lines
☒	☐	Palaeontology and archaeology
☐	☒	Animals and other organisms
☒	☐	Clinical data
☒	☐	Dual use research of concern

Methods

n/a	Involved in the study	
☒	☐	ChIP-seq
☒	☐	Flow cytometry
☒	☐	MRI-based neuroimaging

Animals and other research organisms

Policy information about studies involving animals; ARRIVE guidelines recommended for reporting animal research, and Sex and Gender in Research

Laboratory animals	We bred Emx1-Cre (Jax Stock #005628) × ROSA-LNL-tTA (Jax Stock #011008) × TITL-GCaMP6s (Jax Stock#024104) triple transgenic

Laboratory animals	mice to express GCaMP6s in cortical excitatory neurons. We used 12 to 16 week old mice of both sexes for our experiments (n = 6).
Wild animals	This study did not involve wild animals.
Reporting on sex	All experiments were performed in mice of both sexes. No sex-based analyses were performed.
Field-collected samples	No field-collected samples were used.
Ethics oversight	All animal procedures were approved by the Institutional Animal Care and Use Committee at the University of California, Santa Barbara.

Note that full information on the approval of the study protocol must also be provided in the manuscript.
